# ZBP1 condensate formation synergizes Z-NAs recognition and signal transduction

**DOI:** 10.1038/s41419-024-06889-y

**Published:** 2024-07-09

**Authors:** Feiyan Xie, Di Wu, Jing Huang, Xuehe Liu, Yanfang Shen, Jinqing Huang, Zhipeng Su, Jixi Li

**Affiliations:** 1 Department of Neurology, Huashan Hospital and School of Life Sciences, State Key Laboratory of Genetic Engineering, Fudan University, 200438 Shanghai, China; 2 Department of Parasitology, School of Basic Medical Science, Central South University, Changsha, 410083 Hunan China; 3 Department of Chemistry, The Hong Kong University of Science and Technology, Hong Kong, China; 4 Department of Neurosurgery, First Affiliated Hospital of Wenzhou Medical University, Wenzhou, 325000 China

**Keywords:** Necroptosis, DNA-binding proteins

## Abstract

Z-DNA binding protein 1 (ZBP1) is a crucial player in the intracellular recognition of Z-form nucleic acids (Z-NAs) through its Zαβ domain, initiating downstream interactions with RIPK1 and RIPK3 via RHIM domains. This engagement leads to the assembly of PANoptosomes, ultimately inducing programmed cell death to curb pathogen dissemination. How Zαβ and RHIM domain cooperate to trigger Z-NAs recognition and signal transduction remains unclear. Here, we show that ZBP1 condensate formation facilitates Z-NAs binding and antiviral signal transduction. The ZBP1 Zαβ dimerizes in a concentration-dependent manner, forming characteristic condensates in solutions evidenced by DLS and SAXS methods. ZBP1 exhibits a binding preference for 10-bp length CG (10CG) DNA and Z-RNA ligand, which in turn enhanced Zαβ dimerization, expediting the formation of droplet condensates in vitro and amyloid-like puncta in cells. Subsequent investigations reveal that Zαβ could form condensates with liquid-liquid phase separation property upon HSV and IAV infections, while full-length ZBP1 forms amyloid-like puncta with or without infections. Furthermore, ZBP1 RHIM domains show typical amyloidal fibril characterizations and cross-polymerize with RIPK1 depending on the core motif of _206_IQIG_209_, while mutated ZBP1 could impede necroptosis and antiviral immunity in HT-29 cells. Thus, ZBP1 condensate formation facilitates the recognition of viral Z-NAs and activation of downstream signal transduction via synergic action of different domains, revealing its elaborated mechanism in innate immunity.

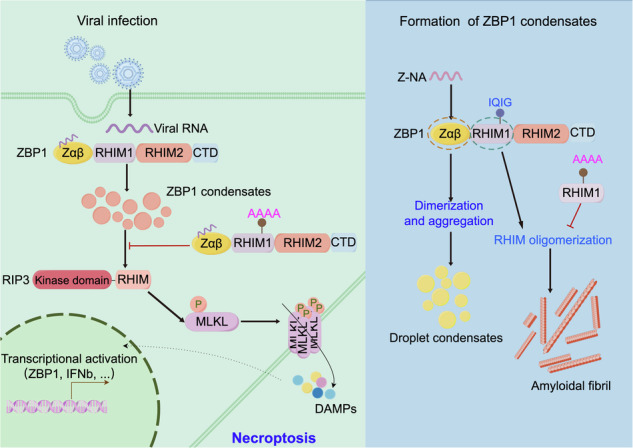

## Introduction

The initiation of innate immunity is anchored on the recognition of various pathogen-associated molecular patterns (PAMPs) or damage-associated molecular patterns (DAMPs) by their corresponding pattern recognition receptors (PRRs) [1]. Among the PRR family, DNA sensors including ZBP1, AIM2, RNA polymerase III, IFI16, DDX41, and cGAS, are critical in identifying exogenous nucleic acid stimuli during pathogen infections, initiating subsequent antiviral and antibacterial immune responses [2–5]. Z-DNA binding protein 1 (ZBP1), also known as DAI or DLM-1, stands out as the pioneering cytoplasmic DNA sensor that detects abnormal DNA and RNA in Z-type conformation (Z-NA) derived from pathogens. Upon recognition, ZBP1 activates multiple downstream signaling pathways, including the release of type I interferon (IFN) and the initiation of programmed cell death [6–8]. This pivotal role of ZBP1 underscores its significance in orchestrating an effective immune response against pathogenic threats.

**Fig. 1:**
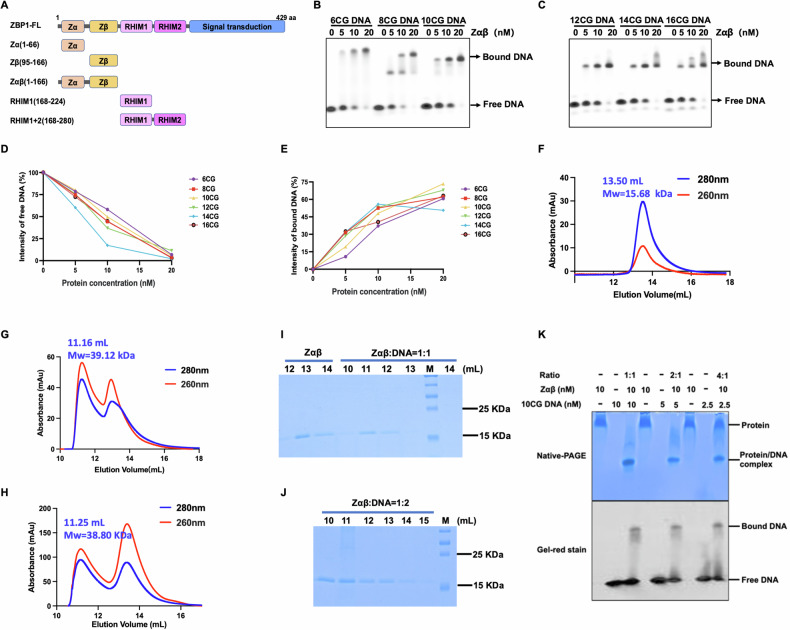
The binding property of ZBP1-Zαβ with unlabeled 10 CG DNA. **A** Schematic of human ZBP1 depicting two Z-NA binding domains (Zα and Zβ), two RHIM domains, and the C-terminal signal transduction domain. **B**, **C** Titration of various lengths of Z-type d(CG)n DNA (*n* = 6, 8, 10, 12, 14, and 16) with Zαβ. Protein concentrations of Zαβ ranged from 0 to 20 nM. **D**, **E** Quantitation of free or bound DNA ligands with Zαβ protein as shown in (**A**) and (**B**). **F**–**H** Gel filtration profiles of ZBP1-Zαβ, ZBP1-Zαβ complexed with 10 CG DNA in a 1:1 ratio, and ZBP1-Zαβ complexed with 10 CG DNA in a 1:2 ratio. **I**, **J** SDS-PAGE of eluted fractions of ZBP1-Zαβ complexed with DNA complex as shown in (**F**)–(**H**). **K** Native-PAGE and gel-red staining of ZBP1-Zαβ with 10 CG DNA.

ZBP1 contains two tandem Z-DNA binding domains (Zαβ) at the N-terminus, which are considered indispensable for ZBP1 activation after Z-NA sensing. Additionally, the intermediate region of ZBP1 encompasses two RIP homotypic interaction motif (RHIM) domains, which form amyloid-like structures in cells [9–11]. The RHIMs facilitate the recruitment of receptor-interacting protein kinase 1 (RIPK1 or RIP1) and RIPK3 (or RIP3), contributing to PANoptosome assembly and instigating programmed cell death as a defense mechanism to restrict pathogen dissemination (Fig. 1A) [12]. ZBP1 is widely involved in antiviral responses, heatstroke, autoinflammatory diseases, and tumorigenesis [13]. However, it is still unclear how ZBP1 is activated in response to Z-NA stimulation, therefore inducing the downstream immune response.

Multiple bioactive proteins adopt varying condensate states through phase separation [14]. Specific proteins form liquid-liquid phase separation (LLPS) or amyloid aggregation to fulfill their biological functions [15]. Intracellular LLPS condensates are often referred to as membrane-less organelles, which are related to various cellular processes, including signal transduction, regulation of gene expression, and stress response [16, 17]. A novel functional mechanism of DNA-mediated condensate formation in LLPS enables cGAS to trigger immune responses upon detecting abnormal longer DNAs over 100 bp [18]. Similarly, NLRP6 could interact with double-stranded RNA (dsRNA) to form LLPS condensates for inflammasome activation, mediating downstream pyroptosis or inflammation [19].

RHIM-containing proteins tend to undergo amyloid aggregation through RHIM-RHIM homotypic interactions, performing distinctive functional and pathological roles [20]. The core motif sequences IQIG (206-209) and VQLG (264-267) in RHIMs of ZBP1 are similar to those in RIPK1 and RIPK3 [9]. Since amyloid aggregation composed of RIPK1 and RIPK3 plays a crucial role in governing necroptosis occurrence, ZBP1 has proved to act as a homeostatic harbor for RHIM-containing RIP kinases, regulating cell death and inflammation [21–23]. The RHIM-dependent interactions between ZBP1 and RIPK1 are instrumental in triggering NF-κB activation, with ZBP1 as an adapter for activation of RIPK3-dependent necroptosis [9, 24]. The Zβ and the first RHIM domain are indispensable for ZBP1-dependent necroptosis [5, 21]. Notably, dysfunction, deficiency, or mutation of RIPK1 within RHIM, triggers ZBP1-dependent inflammation and necroptosis in mice [25, 26].

ZBP1 is a crucial innate immune sensor responsible for detecting viral nucleic acid products, particularly sensing viral transcription to orchestrate host defense against viruses, such as herpes simplex virus 1 (HSV1) and influenza A virus (IAV) [21, 27, 28]. In HSV1 infections, ZBP1 recruits RIPK3 to mediate necroptosis, restricting virus replication in mouse cells [29]. Defective viral genomes (DVGs) produced by IAV could serve as Z-RNA ligands for ZBP1, prompting ZBP1 to initiate cell death pathways within the nucleus of infected cells [28]. Research also suggests that inactive Caspase-6 may serve as a scaffold to enhance interactions between ZBP1 and RIPK3 under IAV infection [30]. Thus, the sensing of viral and endogenous Z-NAs by the Zαβ domain might facilitate ZBP1 condensate formation in response to Z-NAs stimulation, triggering the ZBP1-RIPK3 necrosome formation, thereby activating programmed cell death pathways [31–33].

Here, we reveal that ZBP1-Zαβ prefers to bind with the Z-NA ligands, forming droplet-like condensates both in vitro and in cells. Moreover, the condensates composed of Zαβ and Z-NAs exhibit a non-dynamic manner verified by the fluorescence recovery after the photobleaching (FRAP) assay. Intriguingly, Zαβ forms condensate with liquid-liquid phase separation (LLPS) property upon HSV and IAV infection. In contrast, the RHIM domain and full-length ZBP1 form amyloid-like puncta in cells. Thus, different domains of ZBP1 might function synergistically to defend against viral infections.

## Results

### Human ZBP1 binds with different lengths of Z-DNA

ZBP1 plays an essential role in sensing abnormal nucleic acid in Z-conformation nucleic acids (Z-NAs). Human ZBP1 comprises two Z-NA binding domains (Zα and Zβ), two RHIM domains, and the C-terminal signal transduction domain (Fig. 1A). To investigate how ZBP1 recognizes Z-NAs, we expressed and purified the Zαβ and Zβ domains in *E. coli* (Fig. S1A, S1B). Gel-filtration chromatography showed that human ZBP1-Zαβ and Zβ were eluted out at 88.5 mL and 92.5 mL, respectively, on a Superdex 200 16/600 column, corresponding with molecular weights of 16.69 kDa and 11.49 kDa. This indicates that ZBP1-Zαβ and Zβ were monomers in solution. Double-stranded DNA with (CG)n sequences, referred to as n(CG) (Fig. S1C), is commonly regarded as a general Z-DNA ligand in vivo [34]. Next, we performed a binding assay of ZBP1 with various lengths of 5’-FAM-labeled CG DNA (Fig. 1B, C). The results showed that ZBP1-Zαβ interacted with different lengths of CG DNA (from 6 CG to 16 CG), with a preference for 10 CG DNA evidenced by the EMSA assay (Fig. 1D, E). Furthermore, Zβ showed a similar preference with 10 CG DNA with the lowest intensity of free DNA and the highest intensity of bound DNA (Fig. S1D–S1G). Next, mCherry-tagged ZBP1-Zαβ protein was performed with the same assay, in which mCherry-tagged ZBP1-Zαβ showed binding activities with different lengths of Z-DNA, indicating that the mCherry tag does not affect the interaction (Fig. S2A, S2B).

To exclude the potential influence of the FAM tag and identify the binding ratio between ZBP1 and Z-DNA, the purified ZBP1-Zαβ protein was mixed with various ratio of Z-DNA and then analyzed by gel filtration chromatography and SDS-PAGE (Figs. 1F–J and S1H). ZBP1-Zαβ protein was eluted out at a peak of 13.50 mL on a Superdex75 10/300 column, corresponding with a molecular weight of 15.68 kDa (Fig. 1F). After mixing with 10 CG Z-DNA in a 1:1 or 1:2 molar ratios, Zαβ protein shifted to peaks around 11.16 mL or 11.25 mL, corresponding with the molecular weights of 39.12 kDa or 38.80 kDa, respectively (Fig. 1G–J). The results demonstrated that ZBP1-Zαβ interacted with unlabeled Z-DNA in vitro. Further, SDS-PAGE and gel-staining assays confirmed that ZBP1-Zαβ binds with 10 CG Z-DNA in a 1:1 molar ratio (protein: DNA) (Fig. 1K).

### ZBP1-Zαβ exhibits concentration-dependent dimerization

**Fig. 2:**
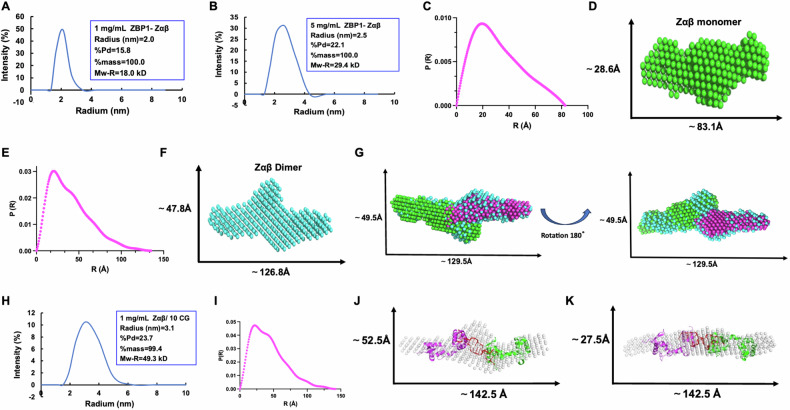
ZBP1-Zαβ dimerizes in a concentration-dependent manner. **A**, **B** The DLS distributions of ZBP1-Zαβ at different concentrations (1 mg/mL: low concentration; 5 mg/mL: high concentration). **C**–**F** The P(R) curve and *de-novo* DAMMIN model of ZBP1-Zαβ at different concentrations (1 mg/mL and 5 mg/mL). **G** Superimposition of two copies of the ZBP1-Zαβ monomer model (low concentration, green and pink) into the ZBP1-Zαβ dimer model (high concentration, cyan). **H** The DLS distribution of ZBP1-Zαβ/10CG DNA complex. **I** The P(R) curve of ZBP1-Zαβ/10CG DNA complex. **J** Predicted structural model of the ZBP1-Zαβ/10CG DNA complex (red: DNA; pink and green: ZBP1-Zαβ) superimposed with the DAMMIN model generated from SAXS (gray). Protein and DNA are shown in cartoon models. **K** The superimposition result viewed with a 90° rotation.

ZBP1 dimerization is essential for its activation, and artificially induced ZBP1 dimerization can promote IFN release without DNA stimulus [35]. Surprisingly, we found that individual ZBP1-Zαβ exhibited a dimeric status at high concentrations, which differs from the monomeric state at low concentrations through the dynamic light scattering (DLS) and Small-angle X-ray scattering (SAXS) analyses. The DLS experiment showed that the radius and estimated molecular weights of ZBP1-Zαβ at 1 mg/mL (denoted as low concentration) and 5 mg/mL (denoted as high concentration) were 2.0 nm and 18.0 kDa, and 2.5 nm and 29.4 kDa, respectively, indicating a propensity for dimerization with increasing concentration (Fig. 2A, B). SAXS results further confirmed this conclusion, as evidenced by that the maximum dimensions (Dmax) and the distance distribution function P(R) for ZBP1-Zαβ at 1 mg/mL and 5 mg/mL were around 82.5Å and 135Å (Fig. 2C, E), respectively. Next, the ab initio envelopes were generated at these concentrations using the *de-novo* DAMMIN model of SAXS (Fig. 2D, F). Also, the ZBP1-Zαβ model at a low concentration exhibited similarity to the anticipated structural configuration of ZBP1-Zαβ predicted by the AlphaFold2 model (Fig. S1I). Moreover, the superimposition of the two models at low concentration was highly consistent with the high-concentration ZBP1-Zαβ model (Fig. 2G), confirming the concentration-dependent dimerization of purified ZBP1-Zαβ. Furthermore, the ZBP1-Zαβ/DNA complex showed an estimated molecular weight of 49.3 kDa, indicating ZBP1-Zαβ might bind with DNA in a 2:1 ratio at high concentration (Fig. 2H). Furthermore, the SAXS results of ZBP1-Zαβ/DNA complex showed a Dmax value of approximately 142.5 Å (Fig. 2I). The ab initio envelope was constructed for the ZBP1-Zαβ/10 CG DNA complex. Moreover, the superimposition of two predicted ZBP1-Zαβ models with the solved 10 CG DNA structure aligned highly consistently with the DAMMIN complex model (Fig. 2J, K), demonstrating that Z-DNA could promote ZBP1-Zαβ dimerization.

### Z-DNA enhances ZBP1 condensates formation in vitro and in cells

**Fig. 3:**
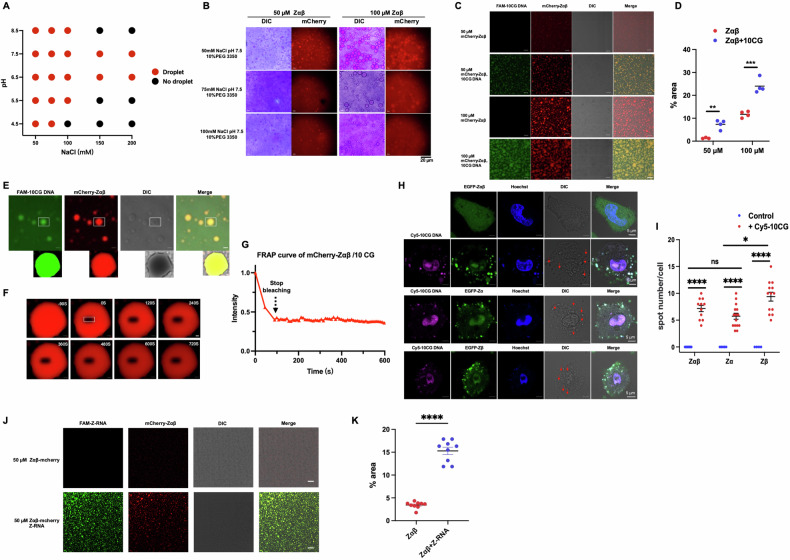
Z-NAs enhance ZBP1 condensates formation in vitro and in cells*.* **A** Phase diagrams illustrating ZBP1-Zαβ condensates formation under a gradient of NaCl concentration and pH conditions. **B** Microscopy images depicting ZBP1-Zαβ condensate formation under various conditions. **C** Confocal images depicting condensate formation composed of ZBP1-Zαβ alone or in complex with 10 CG DNA. Scale bar, 20 μm. **D** Quantification of red droplet-like condensates per field in (**C**). Each dot represents one independent experiment (*n* = 4), and data are presented as mean ± SD. Two-tailed unpaired *t*-test indicated **p < 0.01, ***p < 0.001. **E** Microscopy images showing gel-like condensates formed by purified mCherry-ZBP1-Zαβ protein with FAM-labeled 10 CG DNA. Scale bar, 20 μm. White rectangle indicates zoomed-in droplet for FRAP assay. Scale bar, 2 μm. **F** Images of a mCherry-ZBP1-Zαβ/DNA condensate before and after photobleaching (white box denotes bleach site). Scale bar, 2 μm. **G** Quantitative FRAP curve for mCherry-ZBP1-Zαβ/DNA generated from the bleach site in (**F**). **H** Confocal images showing transfected EGFP-tagged ZBP1-Zαβ, ZBP1-Zα, and ZBP1-Zβ (green) with Cy5-labeled 10 CG DNA (magenta) in HeLa cells. Scale bar, 5 μm. **I** Quantitative analysis of condensates in HeLa cells. Each dot represents the number of condensates in a HeLa cell (*n* > 10); the data are mean ± SD. Two-tailed unpaired *t*-test indicated ns as no significant difference, ***p < 0.001. **J** Confocal images depicting condensate formation composed of ZBP1-Zαβ alone or ZBP1-Zαβ/Z-RNA complex. Scale bar, 20 μm. **K** Quantification of red droplet-like condensates per field in (**J**).

The dimerization characteristic of specific proteins is closely related to their ability to form liquid-liquid phase separation (LLPS) [18, 36]. Next, we explored whether ZBP1-Zαβ could form liquid-liquid phase separation condensates. The mCherry-tagged ZBP1-Zαβ fusion protein was successfully expressed and purified in *E. coli*, and we performed the LLPS screening assay. The result showed that mCherry-ZBP1-Zαβ could form droplet-like condensates under conditions characterized by salt concentration, pH approximating the physiological environment, and 10% PEG 3350 in the solution at different concentrations (Fig. 3A). Further observations under differential interference contrast (DIC) and confocal microscopy showed that ZBP1-Zαβ protein formed typical lipid droplets in vitro under 50–100 mM NaCl, pH 7.5, and 10% PEG3350 (Fig. 3B). Intriguingly, EGFP-tagged ZBP1-Zαβ could not form puncta in HeLa cells, consistent with previous reports [4, 37].

As ZBP1-Zαβ bound with synthetic Z-DNA ligands (Figs. 1 and 2), we further checked whether Z-DNA could promote the LLPS form of ZBP1. The result indicated that mCherry-tagged ZBP1-Zαβ formed droplet condensates with FAM-labeled DNA, and the droplet size increased with the concentration of Zαβ protein (Fig. 3C, D). This suggests that the Z-DNA ligand significantly facilitate the formation of ZBP1-Zαβ condensates. Further fluorescence-recovery-after-photobleaching (FRAP) assay showed that gel-like condensates formed from mCherry-Zαβ protein and FAM-labeled 10 CG DNA did not recover after photobleaching, consistent with individual mCherry-Zαβ condensates (Figs. 3E–G and S2C, S2D). Next, we compared the droplet properties of different Z-DNA binding domains. Following stable expression of EGFP-ZBP1-Zαβ in HeLa cells for 24 h, cells were subsequently transfected with Cy5-10CG DNA, and stained with Hoechst dye after an additional 8 h. While EGFP-ZBP1-Zαβ exhibited a dispersed distribution within cells, it distinctly colocalized with the transfected DNA ligand, forming apparent droplet-like condensates (Fig. 3H). Further investigation revealed that EGFP-ZBP1-Zα or EGFP-ZBP1-Zβ alone could form non-dynamic DNA-induced condensates within cells, with no statistically significant difference in condensate numbers between EGFP-ZBP1-Zαβ, EGFP-ZBP1-Zα, and EGFP-ZBP1-Zβ (Fig. 3H, I). Likewise, all observed condensates were confirmed as non-dynamic, consistent with our in vitro findings (Fig. S3A–S3D). Additionally, endogenous ZBP1 in L929 cells formed puncta after transfection with Cy5-labeled 10 CG DNA (Fig. S3E–S3G). ZBP1 has been proven to interact with Z-RNA produced by IAV [28, 38]. We also observed condensates composed of mCherry-Zαβ with synthetic Z-RNA ligand, and Z-RNA could promote the ability of ZBP1- Zαβ to form non-dynamic condensates (Fig. 3J, K). Thus, distinct from the mechanisms of cGAS or NLRP6, which interact with their specific ligands to form LLPS, our observations indicate that ZBP1-Zαβ and Z-DNA undergo gel-like phase separation, potentially serving as a more stable platform for signal transduction.

### Full-length ZBP1 forms distinct condensates with Zαβ in cells upon virus infection

**Fig. 4:**
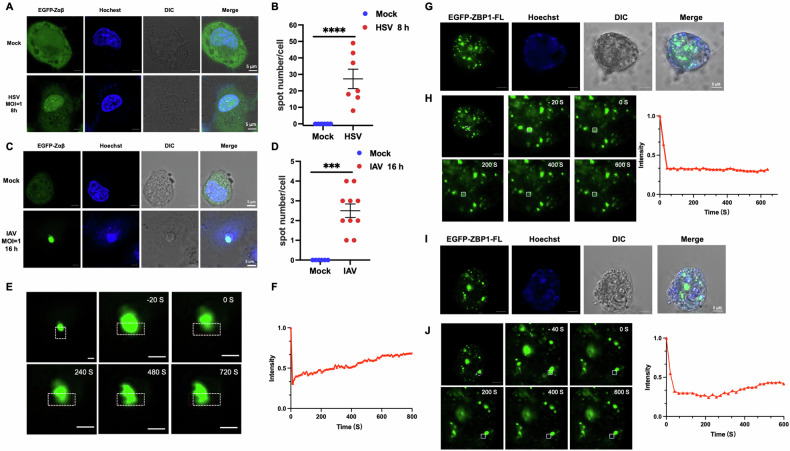
Full-length ZBP1 and Zαβ formed distinct condensates in cells upon virus infection. **A** Confocal microscopy images of transfected EGFP-tagged ZBP1-Zαβ in HeLa cells with or without HSV infection. **B** Quantitative analysis of EGFP-tagged ZBP1-Zαβ condensates in (**A**). Each dot represents the number of condensates in a HeLa cell (*n* = 7), and the data are mean ± SD. Two-tailed unpaired *t*-test indicated ****p < 0.0001. **C** Confocal microscopy images of transfected EGFP-tagged ZBP1-Zαβ in HeLa cells with or without IAV infection. **D** Quantitative analysis of EGFP-tagged ZBP1-Zαβ condensates in (C). Each dot represents the number of condensates in a HeLa cell (*n* = 10), and the data are mean ± SD. Two-tailed unpaired *t*-test indicated ****p < 0.0001. **E** Images of EGFP-tagged ZBP1-Zαβ condensates under IAV infection before and after photobleaching (white box denotes bleach site); stop bleaching event is marked at *t* = 0 s. Scale bar, 5 μm. **F** Quantitative FRAP curve of EGFP-tagged ZBP1-Zαβ condensates under IAV infection generated from the bleach site in (**E**). **G** Confocal microscopy images showing transfected EGFP-tagged full-length ZBP1 in HeLa cells. Scale bar, 5 μm. **H** FRAP images and quantitative curve of EGFP-tagged full length ZBP1 condensates in HeLa cells. Stop bleaching event is marked at *t* = 0 s. Scale bar, 5 μm. **I** Confocal microscopy images of transfected EGFP-tagged full-length ZBP1 upon IAV infection in HeLa cells. Scale bar, 5 μm. **J** FRAP images and quantitative curve of EGFP-tagged full-length ZBP1 condensates upon IAV infection in HeLa cells; stop bleaching event is marked at *t* = 0 s. Scale bar, 5 μm.

To further identify the function of human ZBP1 under pathogens infection, we transfected full-length ZBP1 and Zαβ into HeLa cells, respectively. Notably, ZBP1-Zαβ condensates were observed in the nucleus during infection with DNA virus HSV or RNA virus IAV (Fig. 4A–D). Intriguingly, the FRAP experiment verified that ZBP1-Zαβ condensate showed substantial recovery ability after bleaching during virus infection, indicating that ZBP1-Zαβ could form LLPS with Z-NA derived from viruses (Fig. 4E, F). The different properties of ZBP1-Zαβ condensates might result from its recognition of in vitro synthesized Z-NAs ligands or virus-infected specific Z-NAs. Next, full-length human ZBP1 formed large puncta in HeLa cells upon transfection, with a lack of recovery ability after FRAP assay detection (Fig. 4G, H). Unlike ZBP1-Zαβ, full-length ZBP1 could not form LLPS upon virus infection (Figs. 4I, J and S3H). Considering that ZBP1 contains two amyloid-like RHIM domains besides the Zαβ domain, the distinct condensate properties between full-length ZBP1 and Zαβ domain might result from the RHIM domains, which are conserved with well-known necrosome proteins RIPK1 and RIPK3 [9].

### ZBP1 RHIM domains form amyloid aggregations

**Fig. 5:**
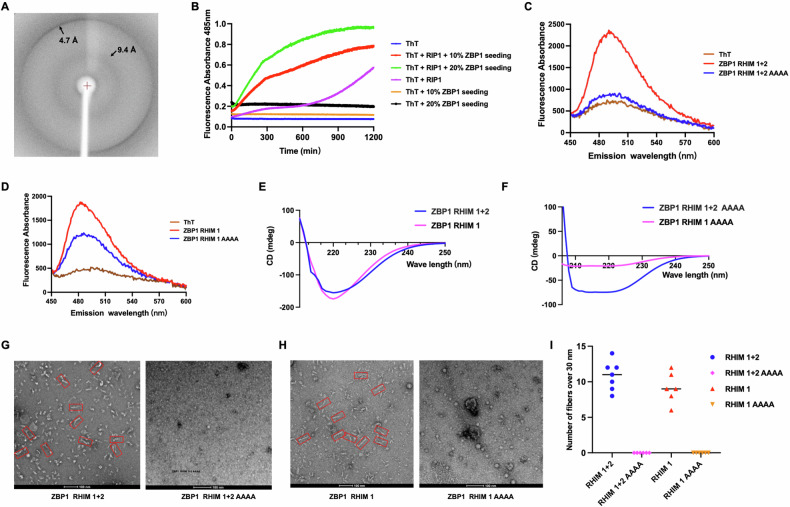
ZBP1 forms amyloid aggregations through RHIM domains. **A** X-ray diffraction image of partially aligned ZBP1 RHIM fibrils. Arrows indicate reflections at 4.7 Å and 9.4 Å resolutions, respectively. **B** Cross-seeding in the polymerization of ZBP1 and denatured RIPK1 assessed using ThT binding assays. **C**, **D** ThT fluorescence emission spectra of wild type ZBP1 RHIMs and their mutants. **E**, **F** CD spectra of wild type ZBP1 RHIMs and their mutants. **G**, **H** Negative-staining EM images showing wild type and 4 A mutant of ZBP1-RHIM 1(Red box indicates representative fibrils over 30 nm in length). **I** Quantitative analysis of representative fibrils in (**G**) and (**H**).

Research has documented the interaction of ZBP1 with other RHIM-containing proteins like RIPK1 and RIPK3 through their shared RHIM domains [9, 26]. To reveal the function of the ZBP1 RHIM domain, we expressed and purified the two RHIMs in *E. coli* (named ZBP1-RHIM 1 + 2, residues 168-280; and ZBP1-RHIM1, residues 168–224). Both constructs formed aggregates eluted out around the void position in the gel-filtration column (Fig. S4A–S4D). We acquired the ZBP1 RHIM 1 + 2 diffraction pattern using Cu radiation, revealing orthogonal diffractions with resolutions of 4.7 Å and 9.4 Å, respectively (Fig. 5A). Amyloid fibrous structures, composed of cross-β strands, were typically identified through the Thioflavin T (ThT) binding experiment. In the presence of RHIM 1 + 2 high-oligomer seed, mixtures containing denatured RIPK1-RHIM proteins showed higher fluorescence absorbances. These data indicate that ZBP1 could cross-polymerize with RIPK1 and form amyloid fibrous structures through RHIM domains (Fig. 5A, B). Furthermore, transfection of the EGFP-tagged RHIM 1 + 2 plasmid into HeLa cells led to the formation of non-dynamic, irregular amyloid aggregations (Fig. S4E–S4G).

To reveal the core motif of ZBP1 RHIM domains responsible for amyloid fibrous structure, the _206_IQIG_209_ sequence was mutated into AAAA (denoted as 4 A) (Fig. S5A–S5D). Although the 4 A mutant protein still came out around the void volume in gel-filtration chromatography, the ThT assay showed a significant decrease in 485 nm fluorescence absorbance intensity for the 4 A mutant (Fig. 5C, D). Additional circular dichroism spectrum revealed the wild-type ZBP1 RHIMs owned a prominent negative peak around 218 nm, but relevant mutants lacked a characteristic peak (Fig. 5E, F). Further, negative-staining electron microscopy (EM) showed that ZBP1-RHIM1 and RHIM1 + 2 formed typical amyloid fibrous structures, with numerous fibrils exceeding 30 nm in length (Fig. 5G, I). However, the 4 A mutants showed fewer and shorter amyloid fibrous structures visualization using negative-staining EM (Fig. 5G, I). Together, ZBP1 forms amyloid aggregations through its RHIM domains, and the _206_IQIG_209_ motif plays an indispensable role in ZBP1 amyloid aggregation formation.

### ZBP1 lacking the IQIG motif impedes necroptosis and antiviral immunity

**Fig. 6:**
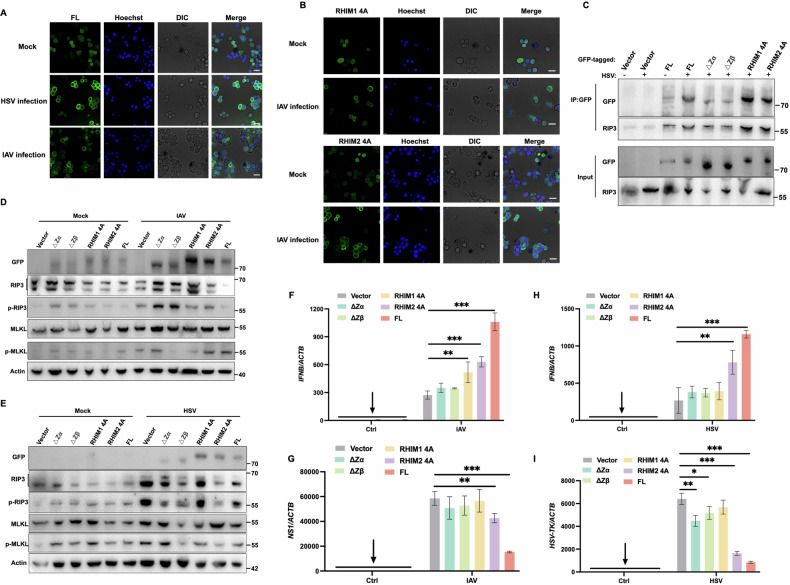
ZBP1 lacking the IQIG motif impedes necroptosis and antiviral immunity. **A** Confocal microscopy images of HT-29 cell lines expressing inducible GFP-tagged wild-type and mutant ZBP1 with IAV or HSV infection (MOI = 1). **B** Confocal microscopy images of HT-29 cell lines expressing different inducible GFP-tagged RHIM motif ZBP1 mutants with IAV infection (MOI = 1). Scale bar, 20 µm. **C** GFP-tagged ZBP1 or its mutants bind with endogenous RIP3. HT-29 cell lysates were immunoprecipitated with an anti-GFP antibody and then immunoblotted with the indicated antibodies. **D**, **E** Representative images of immunoblot analysis of the indicated proteins in HT-29 cells followed by IAV or HSV infection. **F**, **G** qRT-PCR analysis of relative IFNB mRNA and viral RNA level in wild-type and mutant ZBP1 HT-29 cells at 16 h post-infection with IAV. **H**–**I** qRT-PCR analysis of relative IFNB mRNA and viral RNA level in wild-type and mutant ZBP1 HT-29 cells at 8 h post-infection with HSV. (Student’s *t*-test, *p < 0.05, **p < 0.01, ***p < 0.001, and ns, not significant, *n* = 4).

To investigate whether ZBP1 induces necroptosis and inflammation via Zαβ-dependent sensing of Ζ-ΝΑ ligands and RHIM-mediated signaling, we assessed the condensate formation and downstream necrosome formation in response to viral infection using HT-29 cells expressed inducible GFP-tagged wild-type and mutant ZBP1 (Fig. S5A). Fluorescence imaging revealed that ZBP1 expression was upregulated in response to both IAV and HSV infections, forming punctate aggregates (Fig. 6A). This aggregation was significantly reduced following the RHIM1 mutation, whereas the RHIM2 mutation had little effect on ZBP1 aggregation under viral infection conditions (Fig. 6B).

This altered aggregation could impair ZBP1’s ability to effectively participate in necroptosis signaling pathways, which is further verified by the western blotting (WB) and quantitative analyses. Mutations in IQIG could bind to RIP3 confirmed by immunoprecipitation experiment (Fig. 6C), whereas leading to decreased levels of phosphorylated MLKL (Fig. 6D, E). Consequently, cells harboring the RHIM1 mutation exhibited lower cell death rates and diminished LDH release following infection with IAV or HSV (Fig. S5B, S5C). This result suggests that the RHIM1 is crucial for ZBP1’s role in necroptosis, which is a critical process for controlling viral infections and inflammation. By comparing various ZBP1 mutants, we found that only the wild-type full-length ZBP1 was capable of mediating sufficient type I interferon production and inhibiting viral-associated replication (Fig. 6F–I). In summary, our data strongly suggest that the IQIG motif in ZBP1 is essential for its proper functioning in necroptosis and antiviral immunity. The absence of this motif impedes these processes, potentially leading to decreased efficacy in controlling viral infections and regulating inflammation.

## Discussion

ZBP1 serves as a pivotal Z-nucleic acid sensor in the context of antiviral immunity, responding robustly to viruses such as HSV and IAV. Our investigation into the ability of ZBP1 to bind specific DNA ligands and form droplet-like condensates significantly enhances our comprehension of its functional mechanism in Z-NA sensing. We found that ZBP1 Zαβ dimerizes in a concentration-dependent manner, forming characteristic condensates in solutions (Figs. 1 and 2). Additionally, the Zαβ domain possesses the capability to bind Z-RNA, exhibiting analogous characteristics [21]. Interestingly, Zαβ could form condensates with liquid-liquid phase separation properties upon HSV and IAV infections, while full-length ZBP1 formed amyloid-like puncta with or without infections (Figs. 3 and 4). The different properties might result from the additional RHIM domains, which contribute to forming amyloid-like structures in RIPK1 and RIPK3 [9, 10].

Reports showed that the activation of ZBP1 by viral-specific nucleic acids initiates downstream programmed cell death and the release of type I-IFN [30, 39]. Our speculation that extraneous Z-NA induces Zαβ dimerization, creating a platform for ZBP1 self-aggregation and cross-polymerization with other proteins (e.g., RIPK1 and RIPK3) through their RHIM domains, offers insights into the potential of ZBP1 to orchestrate an immune response tailored to various pathogenic infections, while simultaneously averting excessive autoimmune reactions. Moreover, we identified the indispensability of the core IQIG motif in ZBP1 amyloid aggregation formation, elucidating the competitive binding dynamics between RIPK1 and RIPK3 for ZBP1 (Fig. 5), combining the result that mutated ZBP1 lacking the IQIG motif could impede necroptosis induced by the ZBP1/RIPK3/MLKL pathway (Fig. 6) [40]. The observed preference of ZBP1 for binding RIPK1, given their shared IQIG motifs, prompts considerations for a novel drug design strategy aimed at mitigating excessive cell death by targeting these motifs.

Emerging evidence indicating elevated ZBP1 expression levels in tumorigenesis and its involvement in Z-RNA sensing in tumor cells, raising intriguing questions about the potential linkage between the mechanisms underlying ZBP1-Zαβ condensate formation, RHIM amyloid aggregations, and tumor cell death/metastasis. A thorough exploration of this connection can significantly advance our understanding of tumorigenesis and may pave the way for innovative on-therapeutic strategies [31, 41]. In summary, our study not only unravels critical aspects of ZBP1 function in antiviral immunity but also opens avenues for exploring its implications in pathological conditions such as cancer, thus contributing to the broader landscape of immune regulation and disease therapeutics.

## Materials and methods

### Cloning, protein expression, purification, and mutagenesis

The human ZBP1-Zαβ (residues 1-166) was cloned into the pSMT3 vector with a fused 6×His-sumo tag. Plasmids were transformed into *E. Coli* BL21 (DE3) competent cells for protein hyperexpression. *E. coli* cells were cultured in LB medium with 50 μg/mL kanamycin at 37 °C. Strains containing the expression plasmid were induced at OD600 of 0.8 by adding 0.5 mM IPTG, and then continuously grown at 16 °C for an additional 20 h before being harvested at 4 °C. Cells were lysed in a buffer with Tris-HCl (50 mM) at pH 8.0, NaCl (1 M) and imidazole (10 mM). The supernatants of the lysates were incubated with Ni-NTA resin and eluted with a buffer containing Tris-HCl (50 mM) at pH 8.0, NaCl (1 M) and imidazole (250 mM). The 6×His-sumo tag of fusion protein was removed by ULP1 enzyme cleavage overnight at 4 °C. Concentrated target protein was then injected into a Superdex 200 16/600 gel filtration column pre-equilibrated with a buffer containing 20 mM Tris-HCl at pH 7.4 and 100 mM NaCl to collect purified proteins.

All RHIM constructs were expressed in *E. Coli* BL21 Codonplus (DE3)-RIPL cells. Cells were lysed in a solution containing Tris-HCl (50 mM) at pH 8.0, NaCl (1 M), and imidazole (10 mM). The supernatants of the lysates were incubated with Ni-NTA resin, and the target proteins were eluted with a lysis buffer containing a higher concentration of imidazole (250 mM) by centrifugation at 500 rpm. The target protein, without ULP1 digestion, was further applied to a Superdex 200 10/300 gel filtration column pre-equilibrated in a buffer containing 20 mM Tris-HCl and 1 M NaCl buffer at pH 7.5 to acquire purer proteins.

### Z-NA ligands preparation

Six types of 5’-FAM labeled sequences composed of repeated CGs (6CG, 8CG, 10CG, 12CG 14CG, 16CG) and 5’-FAM labeled RNA sequence (ACGCGCGCGCGUUUUUGCGCG-CGCGC) were synthesized (Qingke). DNA sequences were dissolved in a buffer including 100 mM NaCl and 20 mM Tris-HCl at pH 7.4. The single-strand Z-DNA sequences were annealed by heating the solution to 95 °C for 10 min, followed by cooling to 25 °C for 12 h to acquire double-stranded hybridized d(CG)n ligands. 5’-FAM labeled RNA was stabilized by 2’-O-Methyl-8-methyl guanosine (m8Gm; MCE; 2306782-64-7) to form the Z-RNA.

### Dynamic light scattering (DLS)

Dynamic light scattering data were collected and processed using the DYNAMICS software from DynaPro NanoStar (Wyatt Technology). The ZBP1-Zαβ proteins were diluted to 1 mg/mL and 5 mg/mL with 20 mM Tris-HCl (pH 7.4), 100 mM NaCl buffer, and subsequently measured at a light source wavelength of 658 nm and a fixed scattering angle of 90° at room temperature.

### Small-angle X-ray scattering analysis (SAXS)

ZBP1-Zαβ samples at different concentrations (1 mg/mL and 5 mg/mL) in a buffer containing 20 mM Tris-HCl (pH 7.4) and 100 mM NaCl were used for data collection at beamline BL19U2 of the Shanghai Synchrotron Radiation Facility with a radiation wavelength of 1.03 Å. Buffer-alone or protein samples were measured ten times and averaged, and sample measurements were acquired by subtracting the buffer-alone measurements. The ATSAS 2.84 suite was used to process and analyze the ZBP1-Zαβ scattering curve. Radius of gyration (Rg) and I_0_ were determined by the Guinier approximation equation. The GNOM software was used for the evaluation of the maximum particle dimension (Dmax). The DAMMIF program was used to construct ab initio models. The Pymol software was utilized to visualize the model shape.

### Electrophoretic mobility shift assay (EMSA)

ZBP1-Zαβ protein at different concentrations (0, 5 nM, 10 nM, and 20 nM) were mixed with dsDNA ligands (10 nM). The mixtures were incubated on ice for 1 h terminated by adding 2 μL 6× loading buffer (0.05% Bromophenol blue, 0.035% Xylene Cyanol FF, 12% Ficoll 400). Subsequently, the samples were loaded onto a 6% polyacrylamide gel and run for 1 h on ice. The gels were imaged using a Typhoon FLA 9000 machine, and the intensities of the bands were quantified using the ImageQuant TL system.

### Phase separation assay in vitro

The expression and purification of ZBP1-Zαβ with a mCherry tag were conducted following the same procedure as for ZBP1-Zαβ protein. 5 μL of mCherry-ZBP1-Zαβ alone and 5 μL mixture containing a gradient of NaCl concentrations or pH, with 10% PEG 3350 were added to a 96-well plate. The condensates were observed under a confocal microscope (Nikon). FRAP experiments were undertaken on the LSM880 AiryscanTM microscope with a 563 nm laser. Photobleaching was executed with 100% laser power using a 563 nm laser, and images were collected over 10 min after bleaching at 20 s intervals. Fluorescence intensity was measured using ZEN (black edition) v2.3 (Zeiss). The phase-separation assays of protein/Z-NAs complex were undertaken as described above.

### Cellular phase separation assay

After HeLa cells were transfected with pEGFP-N1-Zαβ for 12 h, the cells were transfected with Cy5-labeled DNA using Polyjet. Live-cell images were captured after 8 h using an LSM880 confocal microscope with the 63× oil objective. Images were analyzed by ZEN (black edition) v2.3 (Zeiss). The proteins/Cy5-DNA colocalized condensates in cells was photobleached with 100% laser power for the 488 nm laser, and images were collected over 10 min after bleaching at 20 s intervals.

### Cell culture and transfection

HeLa, L929, and HT-29 cells were grown in DMEM (Gibco) supplemented with 10% FBS (Hyclone) and 100 units/ml penicillin-streptomycin (Hyclone). All cells were cultured at 37 °C in a 5% CO_2_ incubator (Thermo Fisher). ZBP1-Zαβ (1-166), ZBP1-Zα (1-66), and ZBP1-Zβ (95-166) were cloned into pEGFP-N1 vector. HeLa cells were grown on chambered cover glass to an appropriate density and transfected with the respective constructs. In addition, 300 ng/ml doxycycline was placed in a culture solution of HT-29 to induce ZBP1 expression.

### Immunofluorescence

L929 cells were fixed with 4% paraformaldehyde for 10 min at room temperature. Subsequently, the fixed cells were permeabilized using a blocking solution containing 0.1% Triton X-100 and 5% BSA in PBS for 30 min. Primary and secondary antibodies were diluted in PBS to the specified concentrations and incubated for one hour at room temperature. After washing with PBS, cells were mounted with DAPI to counterstain the nucleus and then observed using a Zeiss LSM880 microscope.

### Viral infection

HeLa cells were infected with HSV/IAV (MOI = 1) after plasmid transfection for 24 h, and live cell images were collected at 8 or 16 h after post-infection using an LSM880 confocal microscope with the 63× oil objective. Images were analyzed by ZEN (black edition) v2.3 (Zeiss). HT-29 cells were infected with HSV/IAV (MOI = 1) 24 h after protein expression induction, and live cell images were collected at 8 h or 16 h after infection using a Leica Stellaris 5 WLL confocal with the 63× oil objective. Images were analyzed by LASX office v1.4.6.

### Powder X-ray diffraction

ZBP1-RHIM 1 + 2 was dried and partially aligned by using a SpeedVac System (Savant) and mounted in a cryoloop. The diffraction image was collected by using a Rigaku RU-H3R X-ray generator on a Rigaku R-Axis IV imaging plate detector located at Institute of Organic Sciences, Shanghai.

### Thioflavin T fluorescence

Fluorescence measurements were performed at 25 °C in 96-well plates on a 2300 EnSpire Multimode Plate Reader (Perkin Elmer) with excitation and emission wavelengths of 430 nm and 485 nm, respectively. Thioflavin T fluorescence was used to monitor the fibrillation of ZBP1-RHIM1, ZBP1-RHIM1 + 2, and their mutants in 20 mM Tris, pH 7.4, 100 mM NaCl buffer. The final reaction volume for each experiment was 300 μL, divided into three replicates of 100 μL. The final concentrations of ThT and protein were 25 μM and 5 μM, respectively. At each time point for each experiment, the mean and standard deviation of fluorescence were calculated using all three replicates.

RIP1-RHIM (496-583) was denatured in 8 M urea and then denatured proteins were diluted into a native SEC buffer. The final concentration for the denatured RIP1-RHIM was 10 μM, and ThT was 20 μM. ZBP1-RHIM1 + 2 seeds were added to the reaction at final concentrations were 1 μM, 2 μM, and 3 μM. All reactions were measured kinetically at 25 °C in 96-well plates with excitation and emission wavelengths of 430 nm and 485 nm, respectively.

### Circular dichroism spectrometry

Circular dichroism (CD) spectra of ZBP1 RHIMs and their mutants in a buffer (0.5 mg/mL protein in 100 mM NaCl and 20 mM Tris 7.4) were measured on a Chirascan Plus spectropolarimeter from 190 to 260 nm in a step of 1 nm, employing a cell with a path length of 0.5 mm at 20 °C were obtained. Each spectrum reported is an average of three scans.

### Electron microscopy and image processing

Purified proteins (ZBP1-RHIM1, ZBP1-RHIM1 AAAA, ZBP1-RHIM1 + 2, and ZBP1-RHIM1 + 2 AAAA) were absorbed onto thin carbon films rendered hydrophilic by glow discharge in low-pressure air and supported by copper mesh grids. Samples were washed and blotted with 5 μL of 20 mM Tris at pH 7.4, 100 mM buffer, negatively stained with 5 μL 0.2% uranyl acetate for 60 s, and air dried. Micrographs of negatively stained particles were captured using a 4 K × 4 K Eagle charge-coupled device (CCD) camera on a Tecnai G2 Spirit electron microscope (120 kV).

### Protein preparation and western blotting analysis

Cells were harvested, washed in PBS and lysed in a RIPA lysis buffer (50 mM Tris-HCl, pH 7.5, 150 mM NaCl, 1% Triton X-100 and 1 mM EDTA, 1 mM PMSF). Lytic cells were centrifuged at 17,000 × g for 10 min at 4 °C. Proteins contained in the supernatant were separated by SDS-PAGE, and incubated with specific antibodies: anti-RIP3 (Santa Cruz, sc-374639), anti-MLKL (Abcam, ab184718), anti-p-RIPK3 (Abcam, ab209384), anti-p-MLKL (Abcam, ab187091), anti-GFP (Huabio, ET1607-31), and anti-β-actin (Huabio, ET1702-67).

### Reverse transcription-PCR (RT-PCR)

The total RNA was extracted with TRIzol from HT-29 cell line according to the manufacturer’s instructions (Invitrogen), and was reverse-transcribed into cDNA using the PrimeScript RT Master Mix kit (Takara). Specific primers used for RT-PCR assays were 5’-AGGATTCTGCATTACCTGAAGG-3’, 5’-GGCTAGGAGATCTTCAGTTTCG-3’ for IFNB1; 5’-ATGGATTAGCCATTCAATTCA-3’, 5’-ACCCACGGATGGGACAA-3’ for NS1; 5’-ACCAACTGGGACGACATGGAGAAA-3’, 5’-TAGCACAGCCTGGATAGCAACGTA-3’ for β-actin; The gene expression levels were normalized to those of β-actin.

### Cell viability assays

Cell viability after virus infection was measured using the Cell Counting Kit-8 (APExBIO), while cytotoxicity was determined by the lactate dehydrogenase (LDH) release using LDH Cytotoxicity Assay Kit (Beyotime), then absorbance was measured on the SpectraMax M5 plate reader.

### Statistical analysis

Each experiment was performed at least three times. Data were analyzed using GraphPad Prism 9.0 and presented as the mean ± SD. Statistical analysis was performed using Student’s *t*-test, one-way ANOVA, or two-way ANOVA. Significance levels were denoted as ns (no significant difference), *p < 0.05, **p < 0.01, ***p < 0.001, ****p < 0.0001.

### Supplementary information

Supplementary material

Supplementary Materials-WB raw data

## Data Availability

The SAXS data and model have been deposited in the SASBDB database with accession codes SAS5570 for monomeric ZBP1-Zαβ and SAS5572 for dimeric ZBP1-Zαβ.

## References

[1] Takeuchi O, Akira S (2010). Pattern recognition receptors and inflammation. Cell.

[2] Briard B, Place DE, Kanneganti TD (2020). DNA sensing in the innate immune response. Physiol (Bethesda).

[3] Ha SC, Kim D, Hwang HY, Rich A, Kim YG, Kim KK (2008). The crystal structure of the second Z-DNA binding domain of human DAI (ZBP1) in complex with Z-DNA reveals an unusual binding mode to Z-DNA. Proc Natl Acad Sci USA.

[4] Maelfait J, Liverpool L, Bridgeman A, Ragan KB, Upton JW, Rehwinkel J (2017). Sensing of viral and endogenous RNA by ZBP1/DAI induces necroptosis. EMBO J.

[5] Thapa RJ, Ingram JP, Ragan KB, Nogusa S, Boyd DF, Benitez AA (2016). DAI senses influenza A virus genomic RNA and activates RIPK3-dependent cell death. Cell Host Microbe.

[6] DeAntoneo C, Herbert A, Balachandran S (2023). Z-form nucleic acid-binding protein 1 (ZBP1) as a sensor of viral and cellular Z-RNAs: walking the razor’s edge. Curr Opin Immunol.

[7] Tang Q (2022). Z-nucleic acids: uncovering the functions from past to present. Eur J Immunol.

[8] Balachandran S, Mocarski ES (2021). Viral Z-RNA triggers ZBP1-dependent cell death. Curr Opin Virol.

[9] Li J, McQuade T, Siemer AB, Napetschnig J, Moriwaki K, Hsiao YS (2012). The RIP1/RIP3 necrosome forms a functional amyloid signaling complex required for programmed necrosis. Cell.

[10] Mompean M, Li W, Li J, Laage S, Siemer AB, Bozkurt G (2018). The structure of the necrosome RIPK1-RIPK3 core, a human hetero-amyloid signaling complex. Cell.

[11] Lamour G, Nassar R, Chan PHW, Bozkurt G, Li J, Bui JM (2017). Mapping the broad structural and mechanical properties of amyloid fibrils. Biophys J.

[12] Lee S, Karki R, Wang Y, Nguyen LN, Kalathur RC, Kanneganti TD (2021). AIM2 forms a complex with pyrin and ZBP1 to drive PANoptosis and host defence. Nature.

[13] Karki R, Kanneganti TD (2023). ADAR1 and ZBP1 in innate immunity, cell death, and disease. Trends Immunol.

[14] Alberti S, Gladfelter A, Mittag T (2019). Considerations and challenges in studying liquid-liquid phase separation and biomolecular condensates. Cell.

[15] Banani SF, Lee HO, Hyman AA, Rosen MK (2017). Biomolecular condensates: organizers of cellular biochemistry. Nat Rev Mol Cell Biol.

[16] Ahn JH, Davis ES, Daugird TA, Zhao S, Quiroga IY, Uryu H (2021). Phase separation drives aberrant chromatin looping and cancer development. Nature.

[17] Tsang B, Pritisanac I, Scherer SW, Moses AM, Forman-Kay JD (2020). Phase separation as a missing mechanism for interpretation of disease mutations. Cell.

[18] Hopfner KP, Hornung V (2020). Molecular mechanisms and cellular functions of cGAS-STING signalling. Nat Rev Mol Cell Biol.

[19] Shen C, Li R, Negro R, Cheng J, Vora SM, Fu TM (2021). Phase separation drives RNA virus-induced activation of the NLRP6 inflammasome. Cell.

[20] Riebeling T, Kunzendorf U, Krautwald S (2022). The role of RHIM in necroptosis. Biochem Soc Trans.

[21] Jiao H, Wachsmuth L, Kumari S, Schwarzer R, Lin J, Eren RO (2020). Z-nucleic-acid sensing triggers ZBP1-dependent necroptosis and inflammation. Nature.

[22] Yang D, Liang Y, Zhao S, Ding Y, Zhuang Q, Shi Q (2020). ZBP1 mediates interferon-induced necroptosis. Cell Mol Immunol.

[23] Muendlein HI, Connolly WM, Magri Z, Smirnova I, Ilyukha V, Gautam A (2021). ZBP1 promotes LPS-induced cell death and IL-1beta release via RHIM-mediated interactions with RIPK1. Nat Commun.

[24] Ragonis-Bachar P, Landau M (2021). Functional and pathological amyloid structures in the eyes of 2020 cryo-EM. Curr Opin Struct Biol.

[25] Newton K, Wickliffe KE, Maltzman A, Dugger DL, Strasser A, Pham VC (2016). RIPK1 inhibits ZBP1-driven necroptosis during development. Nature.

[26] Lin J, Kumari S, Kim C, Van TM, Wachsmuth L, Polykratis A (2016). RIPK1 counteracts ZBP1-mediated necroptosis to inhibit inflammation. Nature.

[27] Zheng M, Kanneganti TD (2020). The regulation of the ZBP1-NLRP3 inflammasome and its implications in pyroptosis, apoptosis, and necroptosis (PANoptosis). Immunol Rev.

[28] Zhang T, Yin C, Boyd DF, Quarato G, Ingram JP, Shubina M (2020). Influenza virus Z-RNAs induce ZBP1-mediated necroptosis. Cell.

[29] Shanmugam N, Baker M, Sanz-Hernandez M, Sierecki E, Gambin Y, Steain M (2021). Herpes simplex virus encoded ICP6 protein forms functional amyloid assemblies with necroptosis-associated host proteins. Biophys Chem.

[30] Zheng M, Karki R, Vogel P, Kanneganti TD (2020). Caspase-6 is a key regulator of innate immunity, inflammasome activation, and host defense. Cell.

[31] Karki R, Sundaram B, Sharma BR, Lee S, Malireddi RKS, Nguyen LN (2021). ADAR1 restricts ZBP1-mediated immune response and PANoptosis to promote tumorigenesis. Cell Rep.

[32] Kuriakose T, Man SM, Malireddi RK, Karki R, Kesavardhana S, Place DE (2016). ZBP1/DAI is an innate sensor of influenza virus triggering the NLRP3 inflammasome and programmed cell death pathways. Sci Immunol.

[33] Devos M, Tanghe G, Gilbert B, Dierick E, Verheirstraeten M, Nemegeer J (2020). Sensing of endogenous nucleic acids by ZBP1 induces keratinocyte necroptosis and skin inflammation. J Exp Med.

[34] Herbert A (2019). Z-DNA and Z-RNA in human disease. Commun Biol.

[35] Wang Z, Choi MK, Ban T, Yanai H, Negishi H, Lu Y (2008). Regulation of innate immune responses by DAI (DLM-1/ZBP1) and other DNA-sensing molecules. Proc Natl Acad Sci USA.

[36] Erdel F, Rademacher A, Vlijm R, Tunnermann J, Frank L, Weinmann R (2020). Mouse heterochromatin adopts digital compaction states without showing hallmarks of HP1-driven liquid-liquid phase separation. Mol Cell.

[37] Deigendesch N, Koch-Nolte F, Rothenburg S (2006). ZBP1 subcellular localization and association with stress granules is controlled by its Z-DNA binding domains. Nucleic Acids Res.

[38] Li S, Zhang Y, Guan Z, Ye M, Li H, You M (2023). SARS-CoV-2 Z-RNA activates the ZBP1-RIPK3 pathway to promote virus-induced inflammatory responses. Cell Res.

[39] Guo H, Gilley RP, Fisher A, Lane R, Landsteiner VJ, Ragan KB (2018). Species-independent contribution of ZBP1/DAI/DLM-1-triggered necroptosis in host defense against HSV1. Cell Death Dis.

[40] Ingram JP, Thapa RJ, Fisher A, Tummers B, Zhang T, Yin C (2019). ZBP1/DAI drives RIPK3-mediated cell death induced by IFNs in the absence of RIPK1. J Immunol.

[41] Baik JY, Liu Z, Jiao D, Kwon HJ, Yan J, Kadigamuwa C (2021). ZBP1 not RIPK1 mediates tumor necroptosis in breast cancer. Nat Commun.

