# Supplementary material for: ZBP1 condensate formation synergizes Z-NAs recognition and signal transduction
Source: Cell Death Dis. 2024 Jul 9;15(7):487. doi: 10.1038/s41419-024-06889-y (PMC11233663; doi:10.1038/s41419-024-06889-y)
Supplement: Supplementary file 1 — Supplementary material [file 41419_2024_6889_MOESM1_ESM.docx]

**Supplementary Materials**

**ZBP1 condensate formation synergizes Z-NAs recognition and signal transduction**

Feiyan Xie^1,#^, Di Wu^1,#^, Jing Huang^2^, Xuehe Liu^1^, Yanfang Shen^1^, Jinqing Huang^3^, Zhipeng Su^4,*^, Jixi Li^1,^*

^1^Department of Neurology, Huashan Hospital and School of Life Sciences, State Key Laboratory of Genetic Engineering, Fudan University, 200438, Shanghai, China.

^2^Department of Parasitology, School of Basic Medical Science, Central South University, Changsha, 410083, Hunan, China.

^3^Department of Chemistry, The Hong Kong University of Science and Technology, Hong Kong, China.

^4^Department of Neurosurgery, First Affiliated Hospital of Wenzhou Medical University, Wenzhou, 325000, China.

^#^X.F. and D.W. contributed equally to this work.

*To whom correspondence should be addressed. Email: [lijixi@fudan.edu.cn](mailto:lijixi@fudan.edu.cn) or [drsuzhipeng@wmu.edu.cn](mailto:drsuzhipeng@wmu.edu.cn)

**
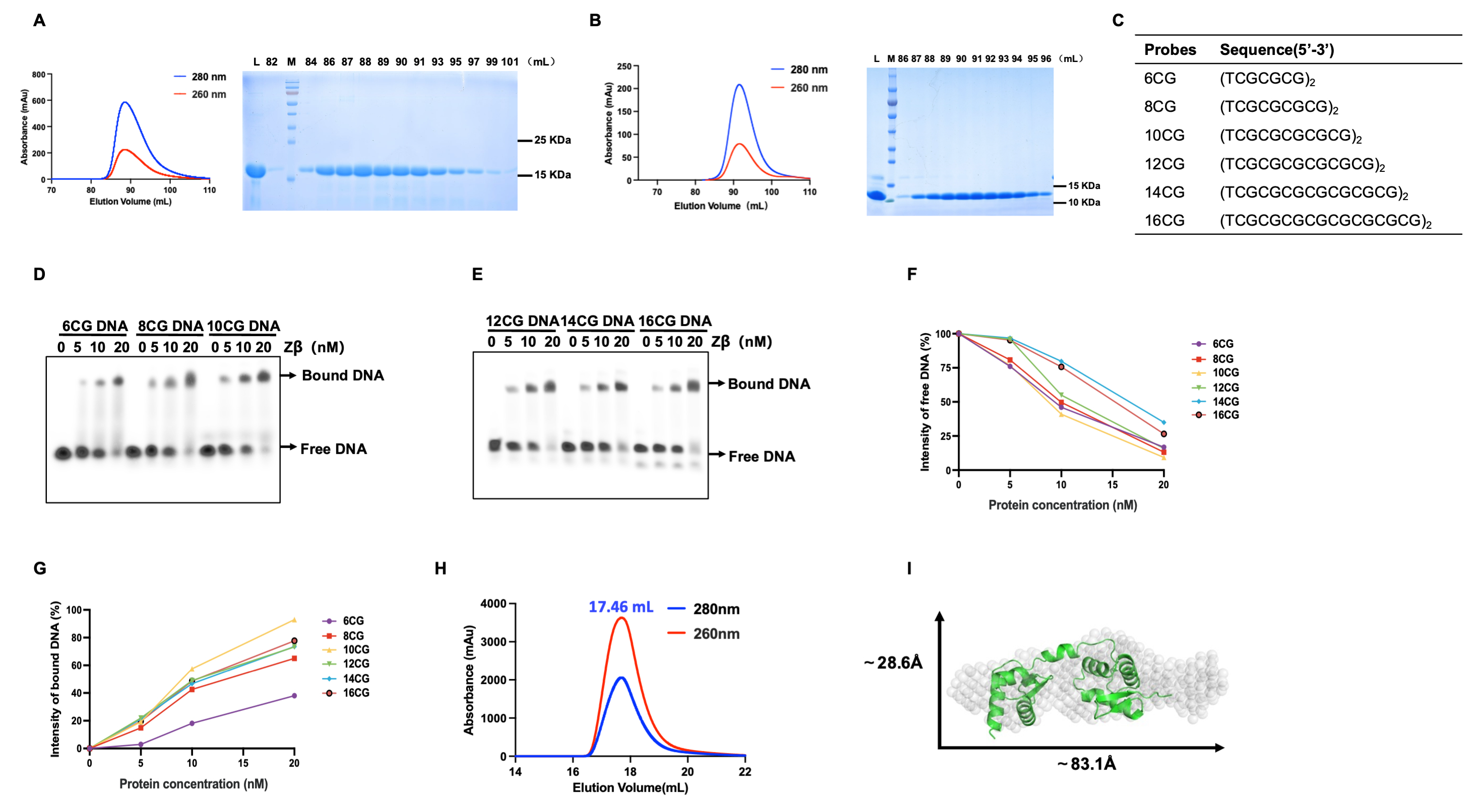
**

**Figure S1. (A-B)** Gel-filtration chromatography profiles and SDS-PAGE results of human ZBP1-Zαβ **(A)** and ZBP1-Zβ **(B)** proteins. Zαβ and Zβ were eluted out at 88.5 mL and 92.5 mL, respectively, on the Superdex 200 16/600 column. L: loading sample; M: protein markers. **(C)** The sequence of synthetic Z-type DNA ligands. (**D-E**) Titration of different lengths of d(CG)n DNA (n=6, 8, 10, 12, 14, and 16) with Zαβ protein. The Zαβ protein concentrations ranged from 0 to 20 nM. (**F-G**) Quantitation of free or bound DNAs with Zαβ protein in panels **D** and **E**. (**H**) Gel filtration profile of the 10 CG DNA alone. **(I)** Predicted structural model of the ZBP1-Zαβ (green) superimposed with the DAMMIN model of purified ZBP1-Zαβ protein at 1mg /mL generated from SAXS (gray).


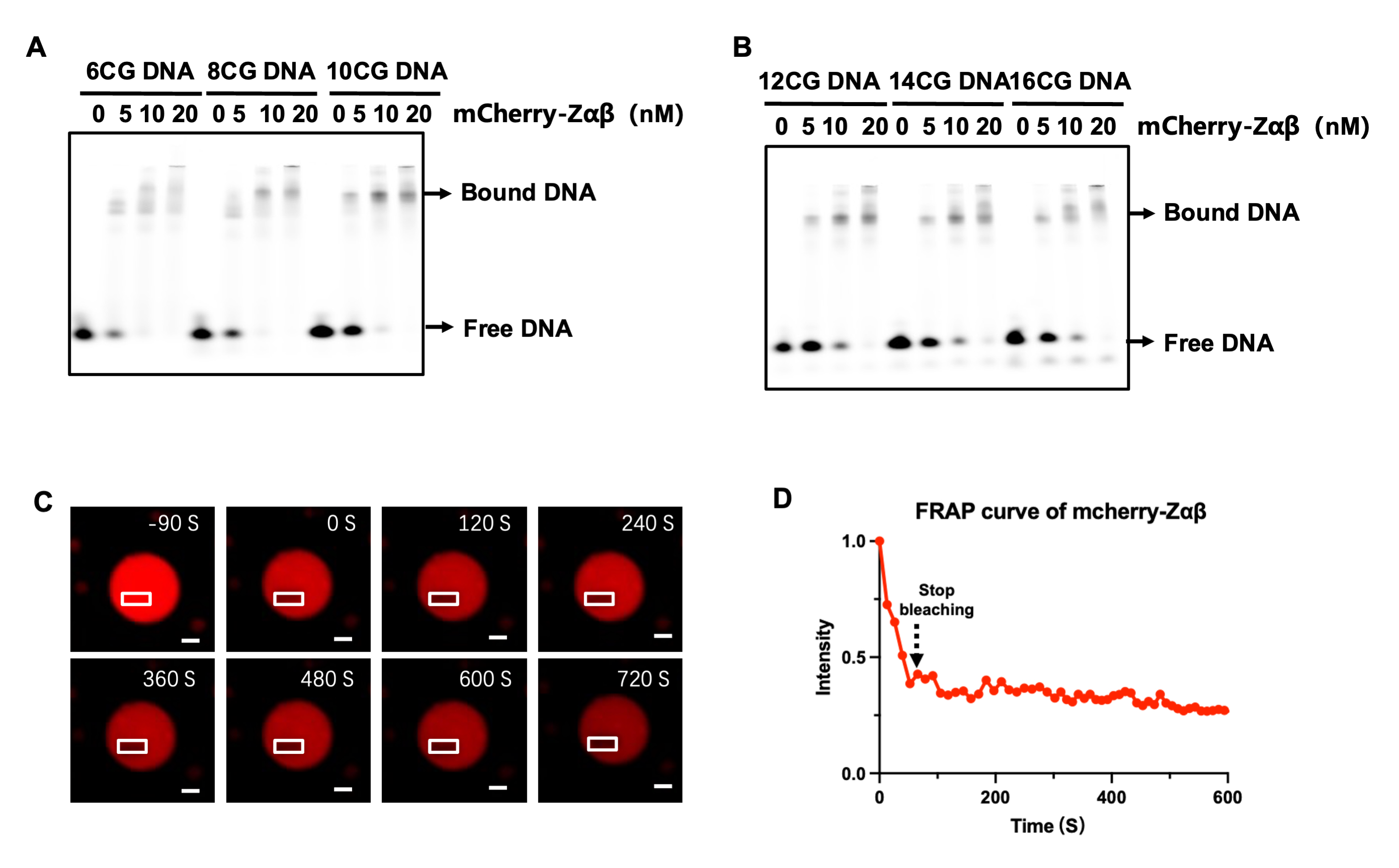


**Figure S2. (A-B)** Titration of different lengths of d(CG)n (n=6, 8, 10, 12, 14, and 16) with human mCherry-Zαβ protein. The concentrations of mCherry-Zαβ fusion protein ranged from 0 to 20 nM. **(C)** Images of a mCherry-ZBP1-Zαβ condensate before and after photobleaching (white box indicates the bleach site). Scale bar, 2 μm. **(D)** Quantitative FRAP curve for mCherry-ZBP1-Zαβ generated from the bleach site in panel **C**.


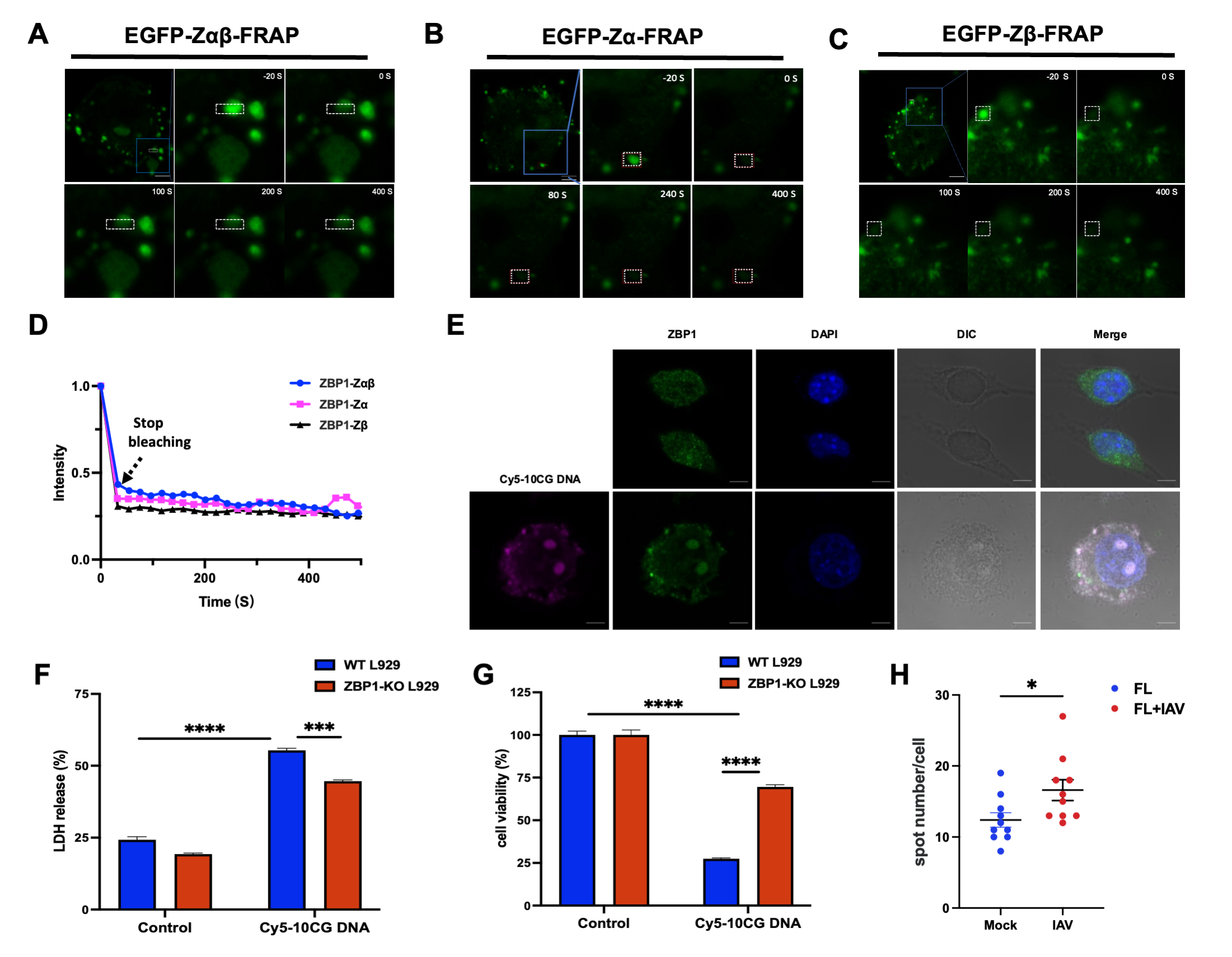


**Figure S3. (A-C)** Condensates composed of EGFP-tagged Zα, Zβ, or Zαβ with DNA ligands were photobleached (white box indicates the bleach site). Scale bar, 5 μm. **(D)** Quantitative FRAP curves generated from bleach sites shown in panels **A-C**. **(E)** Immunofluorescence images of endogenous ZBP1 with or without transfected Cy5-labeled 10 CG DNA in L929 cells. Scale bar, 5 µm. (**F-G**) LDH release and cell viability were detected in the WT or ZBP1 KO L929 cells. Three independent experiments were performed in each group. Statistical analysis was performed using a two-tailed unpaired t-test. ***p < 0.001, ****p < 0.0001. **(H)** Quantitative analysis of EGFP-tagged full-length ZBP1 condensates in HeLa cells with or without IAV infection. Each dot represents condensate numbers in a HeLa cell (n=10), and the data are mean ± SD. Two-tailed unpaired t-test indicated *p < 0.05.


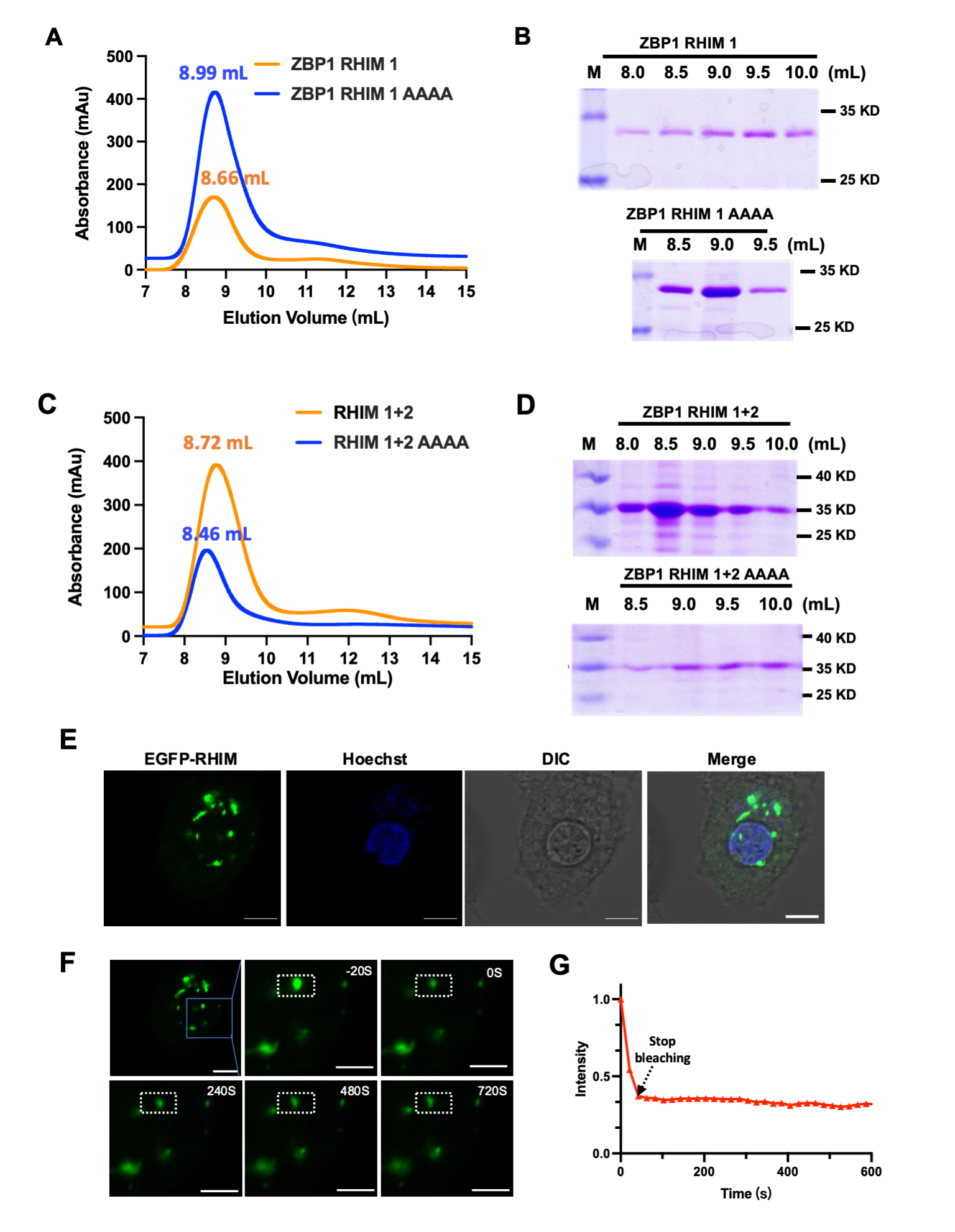


**Figure S4. (A)** Gel filtration profile of ZBP1 RHIM1 and its 4A mutant. **(B)** SDS-PAGE of ZBP1 RHIM1 and its 4A mutant. **(C)** Gel filtration profile of ZBP1 RHIM1+2 and its 4A mutant. **(D)** SDS-PAGE of ZBP1 RHIM1+2 and its 4A mutant. **(E)** Confocal microscopy images of EGFP-tagged ZBP1 RHIM condensates in HeLa cells. Scale bar, 5 μm. **(F)** Images of EGFP-tagged RHIM1+2 condensates before and after photobleaching (white box indicates the bleach site). Stop bleaching event is marked at t=0 s. Scale bar, 5 μm. **(G)** Quantitative FRAP curve generated from bleach site in panel **F**.


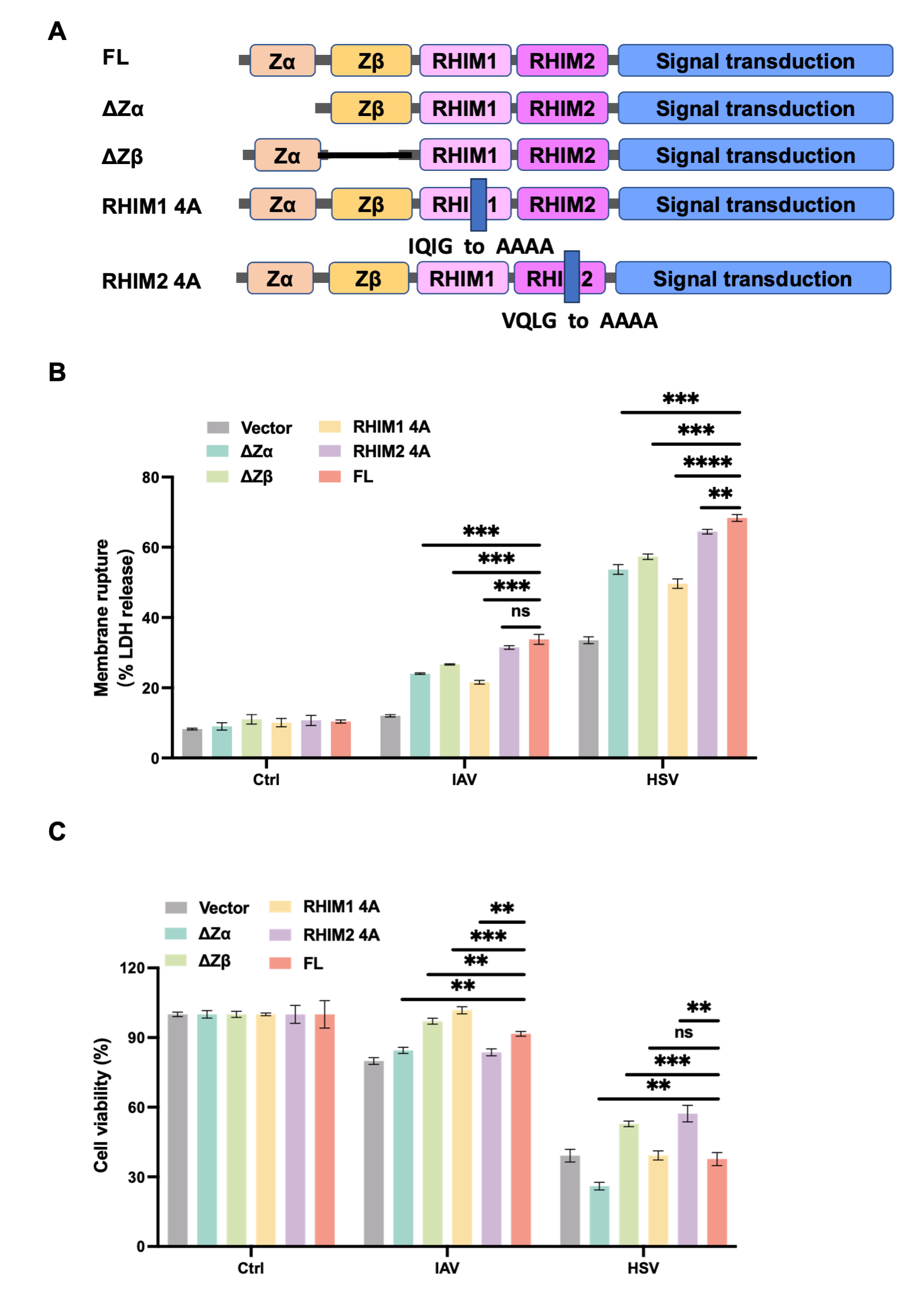
**Figure S5.** (**A**) Schematic of human inducible GFP-tagged wild type and mutant ZBP1. (**B-C**) HT-29 cell lines expressing inducible GFP-tagged wild-type and above mutant ZBP1 were infected with IAV or HSV. Cell viability (**B**) and membrane rupture (**C**) rates were determined by the Cell Counting Kit-8 and the LDH Cytotoxicity Assay Kit, respectively. The data are the means ± SD of triplicate samples from a representative experiment (ns means no significant difference; *p< 0.05; **p < 0.01; ***p < 0.001; ****p < 0.0001). All data are representative of three independent experiments.
