# Supplementary material for: ZBP1 condensate formation synergizes Z-NAs recognition and signal transduction
Source: Cell Death Dis. 2024 Jul 9;15(7):487. doi: 10.1038/s41419-024-06889-y (PMC11233663; doi:10.1038/s41419-024-06889-y)
Supplement: Supplementary file 2 — Supplementary Materials-WB raw data [file 41419_2024_6889_MOESM2_ESM.pptx]

## Slide 1
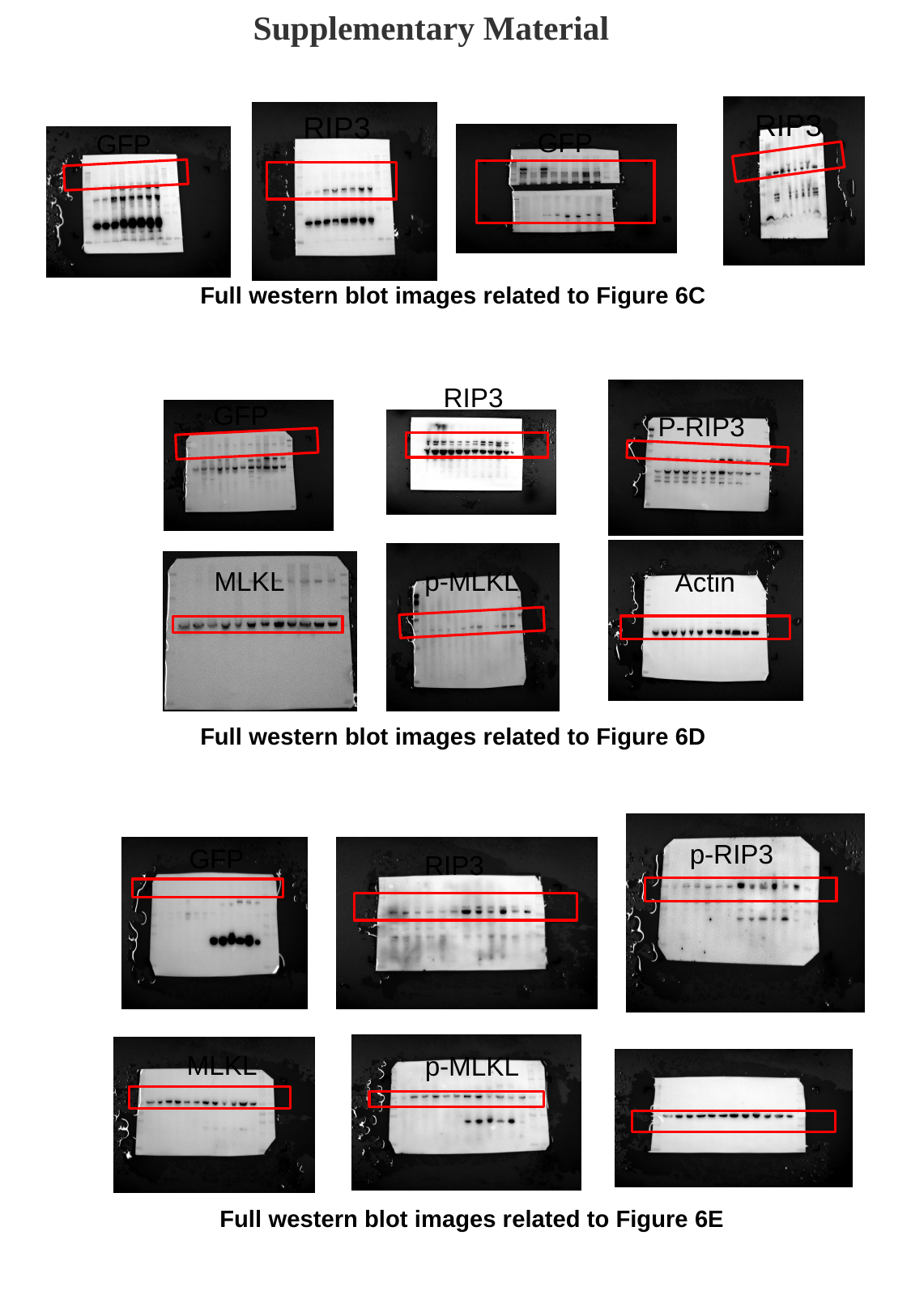

Supplementary Material
RIP3
RIP3
GFP
GFP
Full western blot images related to Figure 6C
RIP3
GFP
P-RIP3
Actin
p-MLKL
MLKL
Full western blot images related to Figure 6D
p-RIP3
GFP
RIP3
MLKL
p-MLKL
Full western blot images related to Figure 6E
